# miR-450b-5p promotes development of endometriosis by inhibiting the GABPA/HOXD10 axis

**DOI:** 10.1016/j.isci.2024.111487

**Published:** 2024-11-28

**Authors:** Yi Huang, Yidan Wang, Ruiyun Li, Yongmei Liu, Yuan Yang

**Affiliations:** 1 The First Clinical Medical College of Lanzhou University, Lanzhou 730000, China; 2 Reproductive Medicine Center, The First Hospital of Lanzhou University, Lanzhou 730000, China; 3 Gansu International Scientific and Technological Cooperation, Base of Reproductive Medicine Transformation Application, Key Laboratory for Reproductive Medicine and Embryo, Lanzhou 730000, China

**Keywords:** Natural sciences, Biological sciences, Molecular biology, Cell biology

## Abstract

Despite decades of research, the pathogenesis of endometriosis remains unclear. Recent studies have shown that microRNAs play an important role in this condition. In this study, we found that the expression level of miR-450b-5p was increased in ectopic endometrial tissues and that GA-binding protein A (GABPA) and HOXD10 expression levels were decreased. Overexpression of miR-450b-5p or knockdown of GABPA significantly promoted the proliferation and invasion of hEM15A cells and inhibited apoptosis *in vivo* and *in vitro*. Furthermore, GABPA was shown to be a direct target of miR-450b-5p and to bind directly to the promoter of the HOXD10 gene, regulating its transcription. Finally, intraperitoneal injection of HOXD10-overexpressing lentivirus in mice significantly attenuated ectopic endometrial lesions. miR-450b-5p directly targets GABPA, regulates expression of HOXD10, and promotes the growth of ectopic endometriotic lesions. Therefore, the miR-450b-5p/GABPA/HOXD10 signaling pathway may be a potential target for treatment of heterotopic endothelial cell disease.

## Highlights

• HOXD10 was transcriptionally upregulated by GABPA
• GABPA was downregulated by miR-450b-5p
• MiR-450b-5p relied on GABPA to inhibit the expression of HOXD10
• Disrupting the miR-450b-5p/GABPA/HOXD10 signaling axis could inhibit endometriosis

## Graphical abstract

**Figure:**
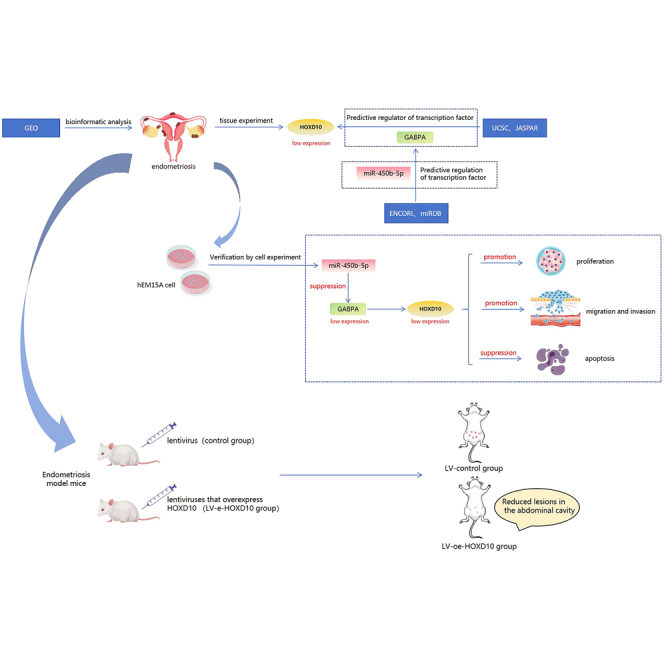

## Introduction

Endometriosis (EMS) is a non-malignant gynecological disease characterized by ectopic growth of endometrial tissue, affecting 5–10% of women of reproductive age worldwide.^1^ Infertility and dysmenorrhea are common clinical manifestations. Because the pathogenesis is not clear, the early diagnosis and treatment of EMS are stuck in a bottleneck. The pathogenesis of EMS involves many aspects such as heredity, environment, infection, and immunity.^2^ Although there are a variety of mechanism models to explain the occurrence of EMS, the exact pathogenesis has not been known so far. Among them, retrograde menstruation is widely accepted as one of the models, the main reason is that retrograde menstruation does occur frequently in EMS.^3^ Studies have shown that abnormal hormonal changes in the body, the role of inflammatory factors, immune disorders, genetic and epigenetic factors, and environmental factors are important reasons for the development of EMS, and the endometrium is greatly affected by hormones, especially estrogen, during menstruation in primates.^4^^,^^5^ Endometrial stromal cells have been widely used in the study of endometrial EMS, and the proliferation and ectopic growth of endometrial stromal cells is one of the main entry points for the pathogenesis of endometrial EMS. Current studies have proved that epigenetics plays a role in the occurrence and development of EMS.^6^^,^^7^ Epigenetic modifications are chemical or physical modifications that affect gene function and thus regulate gene reading and expression without altering the nuclear DNA sequence. These changes are both heritable and reversible, and are a key factor in the progression of EMS. They act as a catalyst for the invasive spread of cells.

MicroRNAs (miRNAs) are a class of non-coding RNAs that regulate gene expression post-transcriptionally by partially binding to target mRNAs and participate in various activities at the cell level.^8^^,^^9^^,^^10^ miRNAs can inhibit or promote cancer by targeting oncogenes or suppressor genes. The study of miRNA has provided a new perspective for understanding how tumors develop and led to further ideas in terms of their diagnosis, treatment, and prognosis.

The normal function of miRNAs being abnormally inhibited is one of the significant factors in the occurrence of EMS. Zhou et al.^11^ found that miR-205-5p can directly target ANGPT2 and indirectly regulate the AKT/ERK signaling pathway, playing an inhibitory role in EMS. The expression of miR-370-3p tends to be downregulated in EMS, which can inhibit the proliferation, metastasis, and invasion of hEM15A cells and promote cell apoptosis. Li et al.^12^ found that miR-92a inhibits the development of EMS by inhibiting the expression of PTEN. *In vitro* experiments confirmed that miR-92a, through antagomir inhibition, can enhance the therapeutic effect of progesterone, thereby inhibiting stromal cell proliferation and reducing the formation of ectopic lesions in mouse models of EMS. This suggests that miR-92a may be a key regulator of EMS proliferation and metastasis. In our research, we have focused on miR-450b-5p based on the results of chip analysis. According to the miRBase database, the total length of miR-450b-5p is 22 nucleotides. To date, studies of miR-450b-5p have been confined to rhabdomyosarcoma and corneal eye disease, with none on the role of miR-450b-5p in EMS.^13^^,^^14^ Therefore, the aim of this study was to identify the mechanism via which HOXD10 influences the invasiveness of EMS in the hope of establishing a basis for identifying new therapeutic targets.

Our study discovered that miR-450b-5p is upregulated in ectopic endometrial tissue, while GABPA and HOXD10 are downregulated. We demonstrated that miR-450b-5p suppresses GABPA, leading to decreased HOXD10 expression, which in turn promotes cell proliferation and invasion and inhibits apoptosis, contributing to EMS. Bioinformatics and a luciferase reporter assay confirmed miR-450b-5p’s direct targeting of GABPA. Western blotting and RT-qPCR further validated this regulation at both protein and mRNA levels. Our findings suggest that the miR-450b-5p/GABPA/HOXD10 axis could be a promising therapeutic target for EMS.

## Results

### Downregulation of HOXD10 was correlated with a poor prognosis

**Figure 1:**
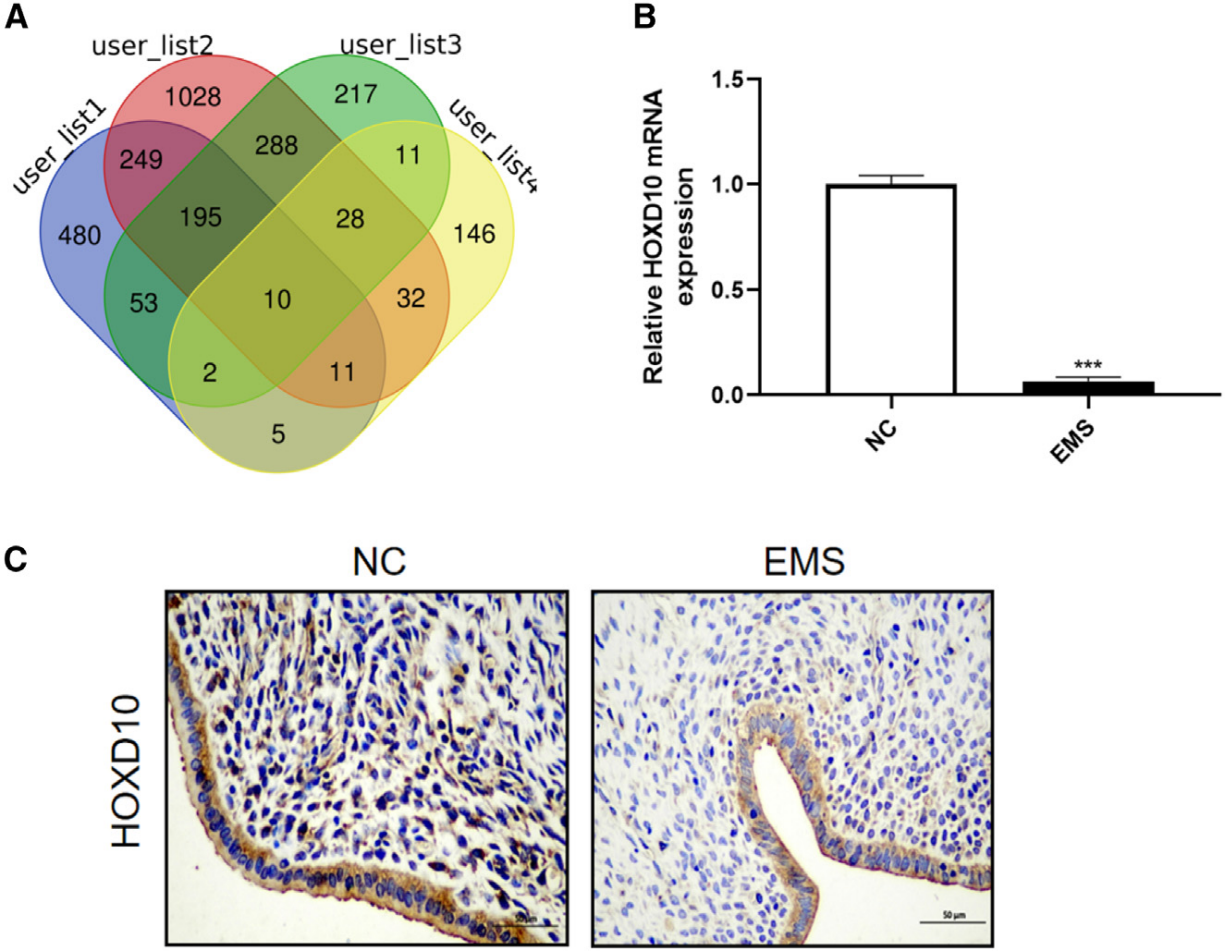
HOXD10 is low expressed in EMS (A) Upregulated and downregulated differentially expressed genes were extracted from four GEO datasets: GSE5108, GSE7305, GSE23339 and GSE58178, respectively, and intersection differentially expressed genes were extracted by Wayne diagram. (B) The expression of differential genes in normal and ectopic endometrial tissues of mice was detected by RT-qPCR. The results showed that HOXD10 expression decreased in the EMS group compared with the control group (∗∗∗*p* < 0.001). (C) Immunohistochemical results showed that HOXD10 expression was low in EMS patients (*p* < 0.05).

We obtained the GEO EMS dataset using the Xiantao Academic Bioinformatics tool, which synthesizes data from multiple transcriptome databases. The necessary datasets were retrieved using “endometriosis” as the keyword, “Homo sapiens,” as the search condition, and “chip” as the GEO2R type to be analyzed. Finally, the following four GEO datasets were selected: GSE5108, GSE7305, GSE23339, and GSE58178. Differentially expressed genes that were upregulated or downregulated were extracted from these datasets and their intersections identified. The screening condition was |log2 fold-change| >1. After correction, *p* < 0.05. The fold-change was used to extract the intersecting differentially expressed genes, which were then visualized using a Wayne diagram (Figure 1A). The intersecting differentially expressed genes were screened to identify those that were not previously linked with EMS. Next, RT-qPCR was performed to confirm the differentially expressed genes to be included in our mouse model of EMS.

We found that the average expression of HOXD10 mRNA was more than three times lower in ectopic endometrial tissues than in normal control tissues (Figure 1B). Immunohistochemistry also showed that HOXD10 expression was significantly lower in ectopic lesions from patients with EMS than in normal human endometrial tissues (*p* < 0.05) (Figure 1C).

### HOXD10 inhibited cell growth, migration, and invasion but promoted apoptosis in hEM15A cells

**Figure 2:**
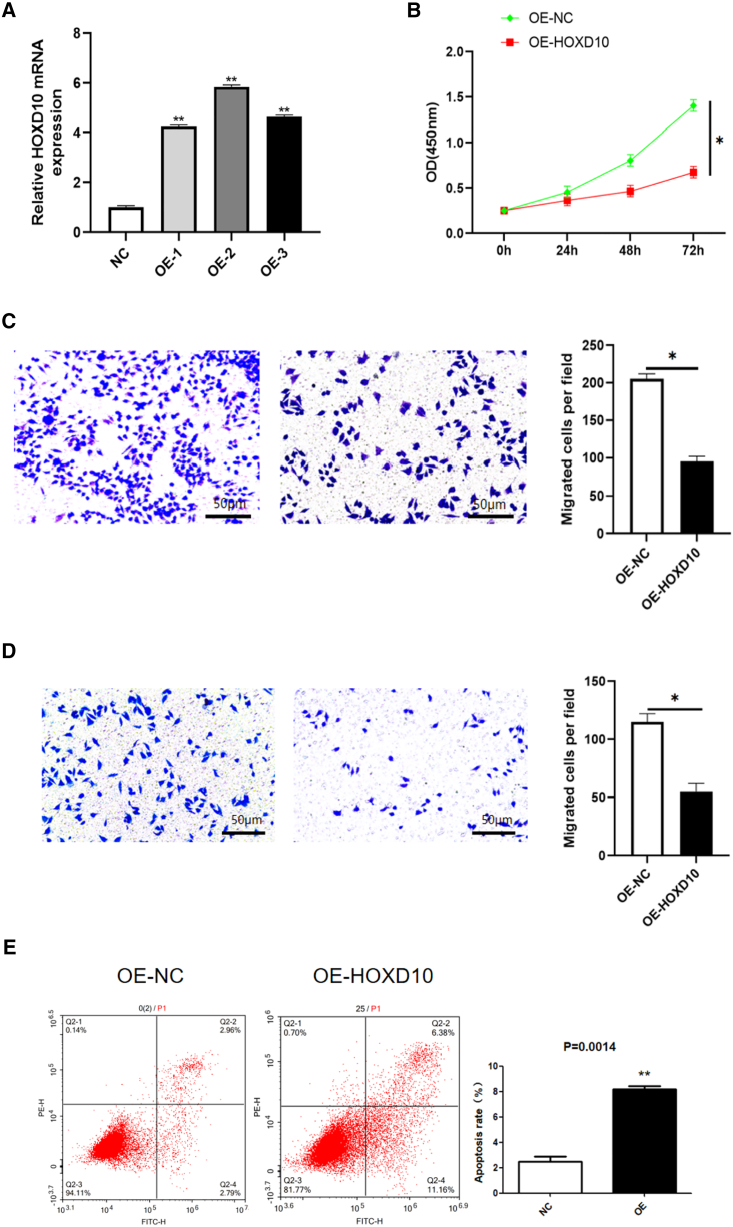
Effect of overexpression of HOXD10 on hEM15A (A) RT-qPCR verified the overexpression efficiency of HOXD10, and the results showed that the expression of HOXD10 in OE-1(overexpression of HOXD10-1), OE-2(overexpression of HOXD10-2) and OE-3(overexpression of HOXD10-3) was more than three times(∗∗*p* < 0.01). (B) The proliferation ability of hEM15A after OE-HOXD10 was detected by CCK-8 assay, and the results showed that the proliferation ability of OE-HOXD10 group was significantly decreased compared with the control group(∗*p* < 0.05). (C and D) Transwell detected the migration and invasion ability of hEM15A after OE-HOXD10, and the results showed that the migration and invasion ability of OE-HOXD10 group was significantly lower than that of control group (∗*p* < 0.05). (E) Annexin V/PI double staining+flow sorting to detect the apoptosis of hEM15A after OE-HOXD10. The results showed that the apoptosis rate of OE-HOXD10 group was increased compared with the control group (∗∗*p* < 0.01).

Next, we investigated changes in proliferation, migration, invasion, and apoptosis in hEM15A cells after overexpression of HOXD10. RT-qPCR confirmed that HOXD10 expression was more than three times higher in hEM15A cells that overexpressed HOXD10 (the OE- HOXD10 group) than in normal control cells that did not overexpress HOXD10 (the OE-NC group); this finding was statistically significant. HOXD10 expression was more than 3x higher in cells that were transfected with plasmids to over-express HOXD10 (Figure 2A). We selected the OE-2 (overexpression of HOXD10-2) group for the next experiment. Cell Counting Kit (CCK)-8 assays revealed that optical density increased more slowly in the OE-HOXD10 group than in the OE-NC group, indicating that proliferation of hEM15A cells was significantly inhibited by HOXD10 overexpression (Figure 2B). Transwell migration and invasion assays showed that transfection with OE-HOXD10 significantly inhibited migration and invasion of hEM15A cells in comparison with the control cells (Figures 2C and 2D). Annexin V/propidium iodide (PI) double staining and flow cytometry showed that a high proportion of Annexin V-positive cells promoted apoptosis in the OE-HOXD10 group (Figure 2E). Collectively, these findings confirmed that HOXD10 alters cell behavior and has a role in proliferation of endometrial cells.

### HOXD10 was transcriptionally upregulated by GABPA

**Figure 3:**
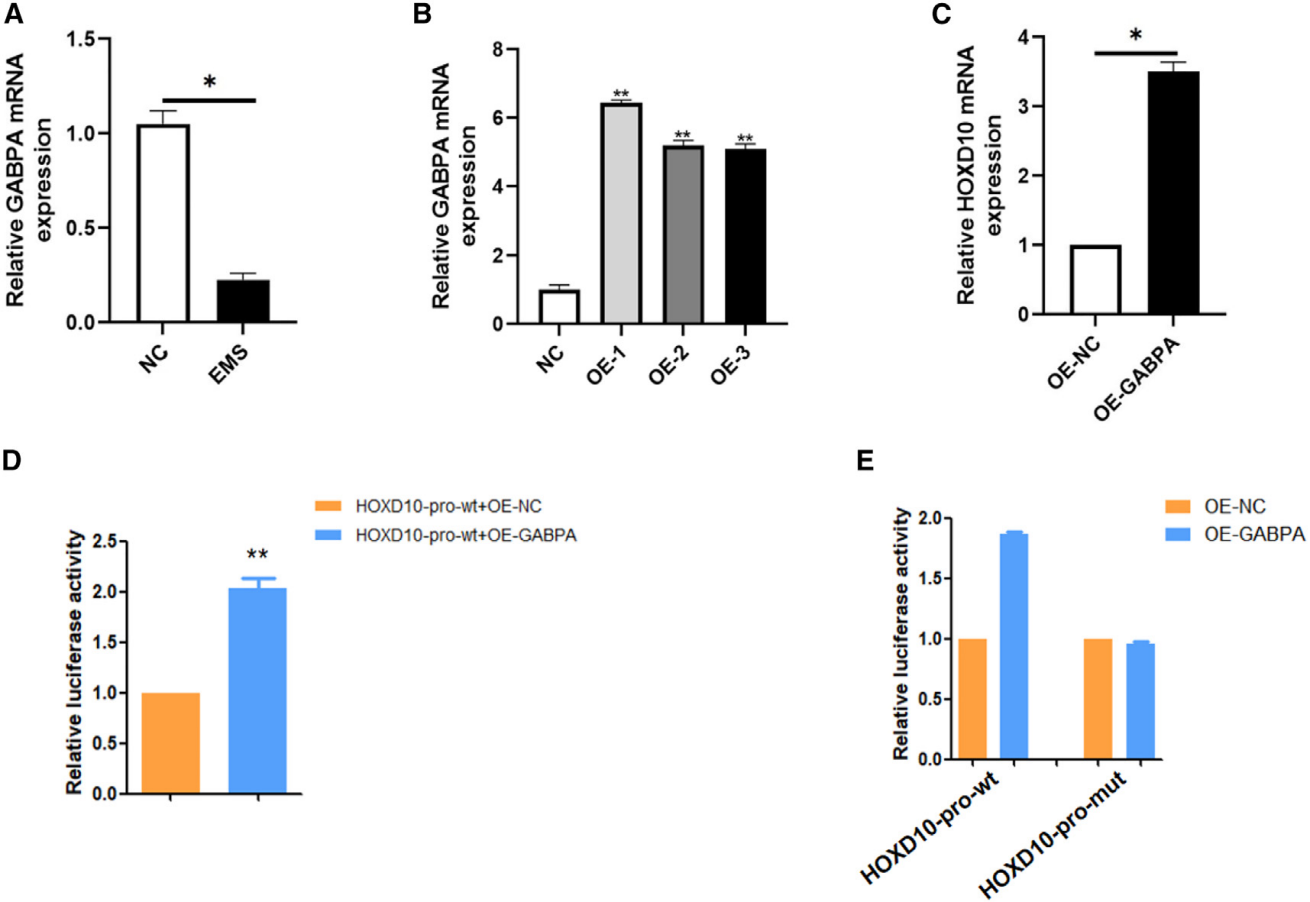
HOXD10 is transcriptionally upregulated by GABPA (A) The expression of GABPA in normal and ectopic endometrial tissues of mice was detected by RT-qPCR. The results showed that GABPA expression decreased in EMS group compared with control group (∗*p* < 0.05). (B) RT-qPCR verified the overexpression efficiency of GABPA, and the results showed that the expression of GABPA in OE-1(overexpression of GABPA-1), OE-2 (overexpression of GABPA-2) and OE-3(overexpression of GABPA-3) was more than three times than the control group (∗∗*p* < 0.01). (C) RT-qPCR verified the overexpression efficiency of GABPA, and the results showed that the expression efficiency of OE-GABPA group was more than 3 times that of NC group(∗*p* < 0.05). (D and E) Double luciferase reporter gene assay showed that GABPA regulates HOXD10 transcription (∗∗*p* < 0.01).

Having demonstrated that HOXD10 is a crucial inhibitory factor in EMS, we then explored its upstream regulation. We selected a region of approximately 2 kb near the transcription start site for HOXD10 and used the Jaspar TFBS hub of UCSC to locate transcription factors. We identified GABPA to be a transcription factor that could potentially regulate HOXD10. RT-qPCR showed low expression of GABPA in ectopic endometrial tissues (Figure 3A). Subsequently, GABPA was overexpressed, and OE-1(overexpression of GABPA-1),OE-2(overexpression of GABPA-2) and OE-3(overexpression of GABPA-3) all reached overexpression rates, with significant *p*-values (Figure 3B). RT-qPCR showed that the HOXD10 expression level was significantly increased when GABPA was overexpressed (Figure 3C). The effect of GABPA on transcriptional activation of HOXD10 was evaluated using a double luciferase reporter assay. The results showed that GABPA bound to the promoter region of HOXD10 and promoted transcription of the luciferase reporter gene, resulting in a corresponding increase in the biofluorescence emitted by the luciferase-catalyzed substrate (Figure 3D). GABPA was shown to bind to the promoter region of HOXD10 for transcriptional activation (Figure 3E).

### GABPA inhibited cell growth, migration, and invasion but promoted apoptosis via HOXD10

**Figure 4:**
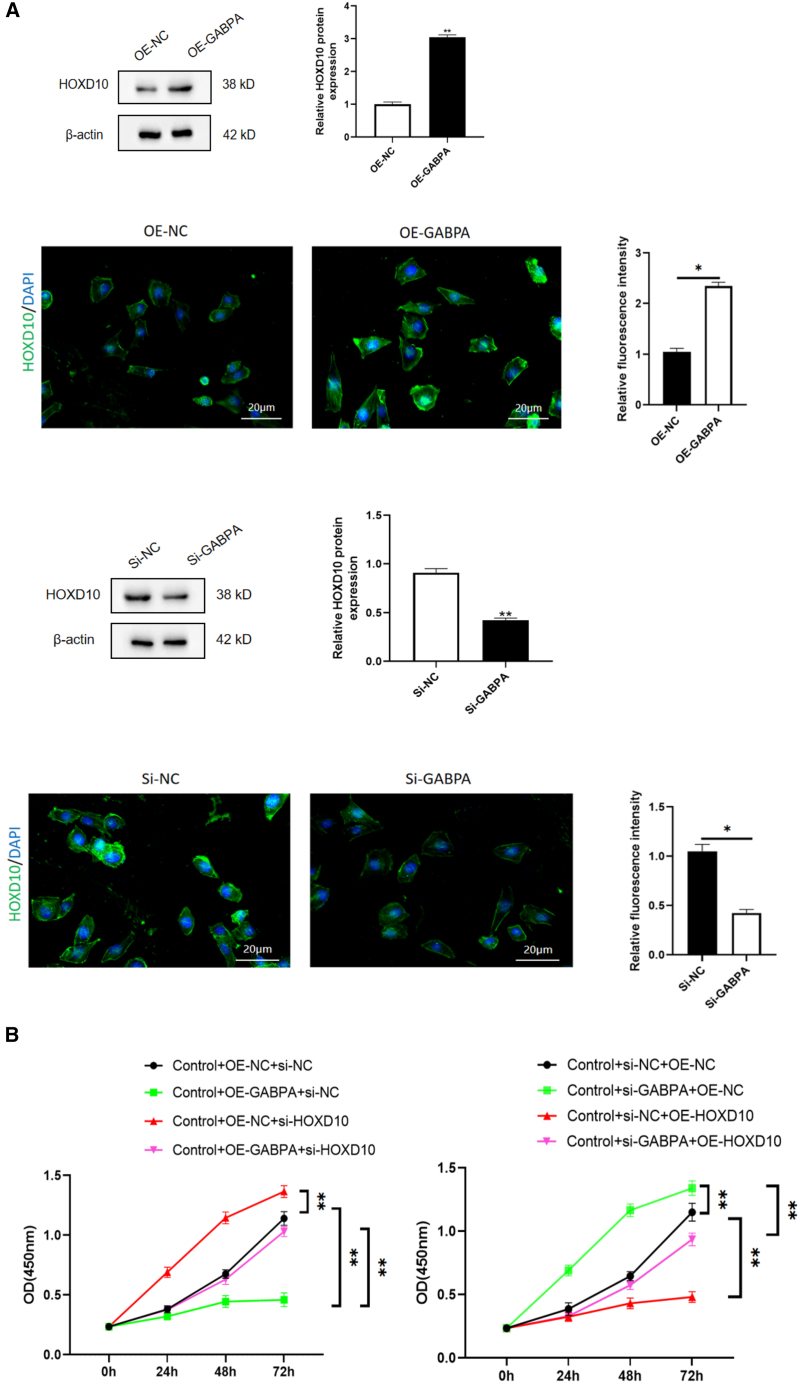

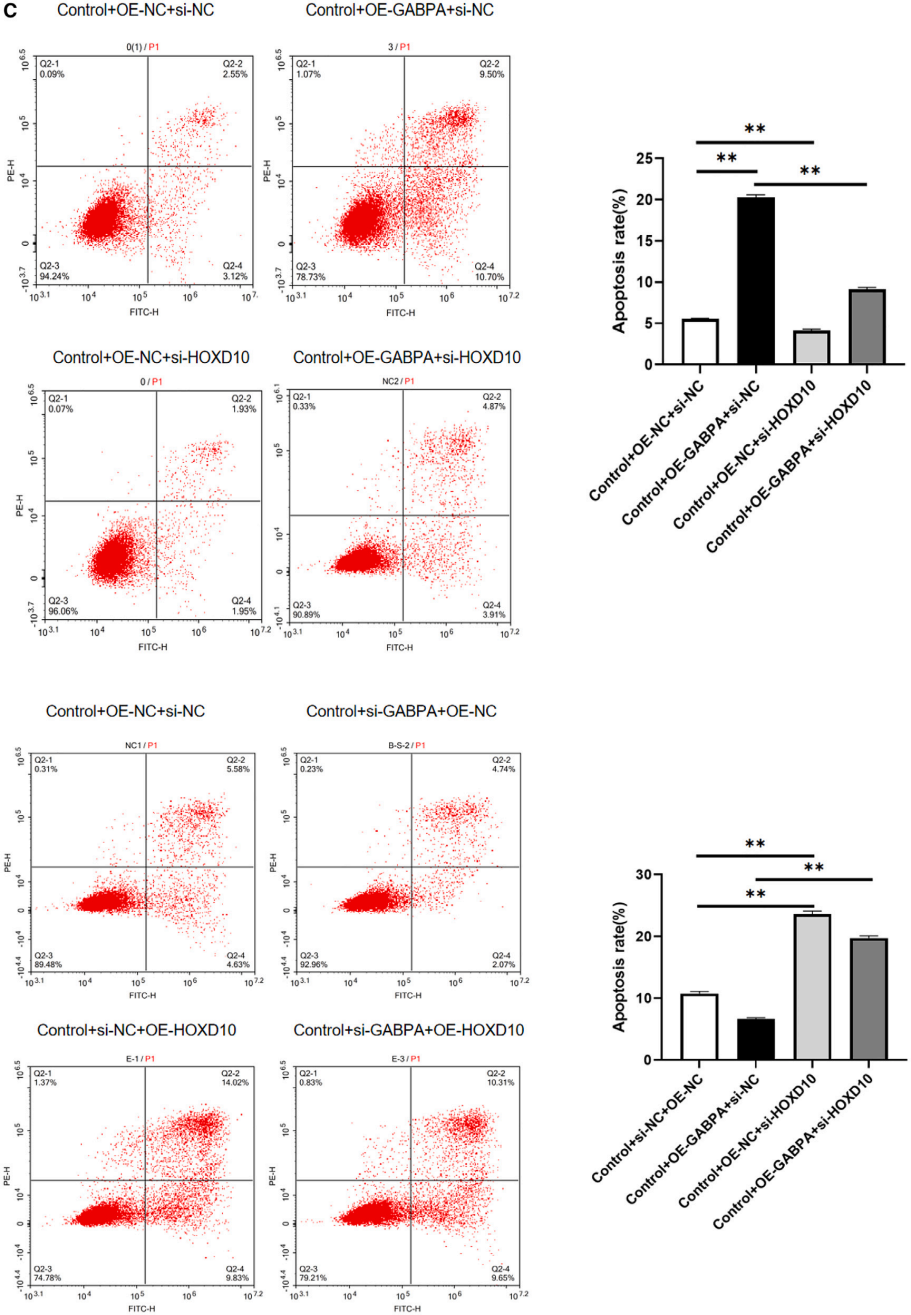

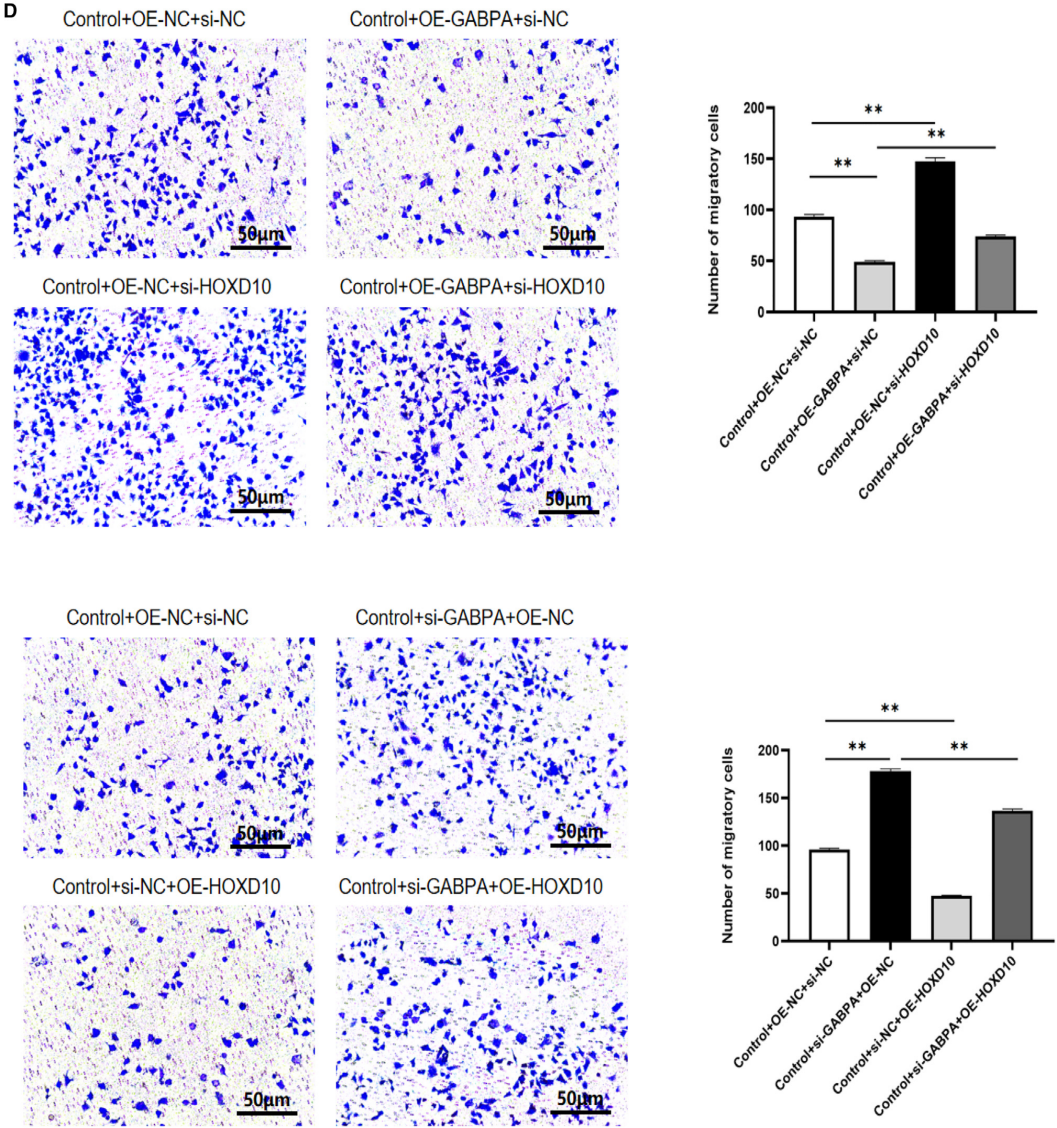

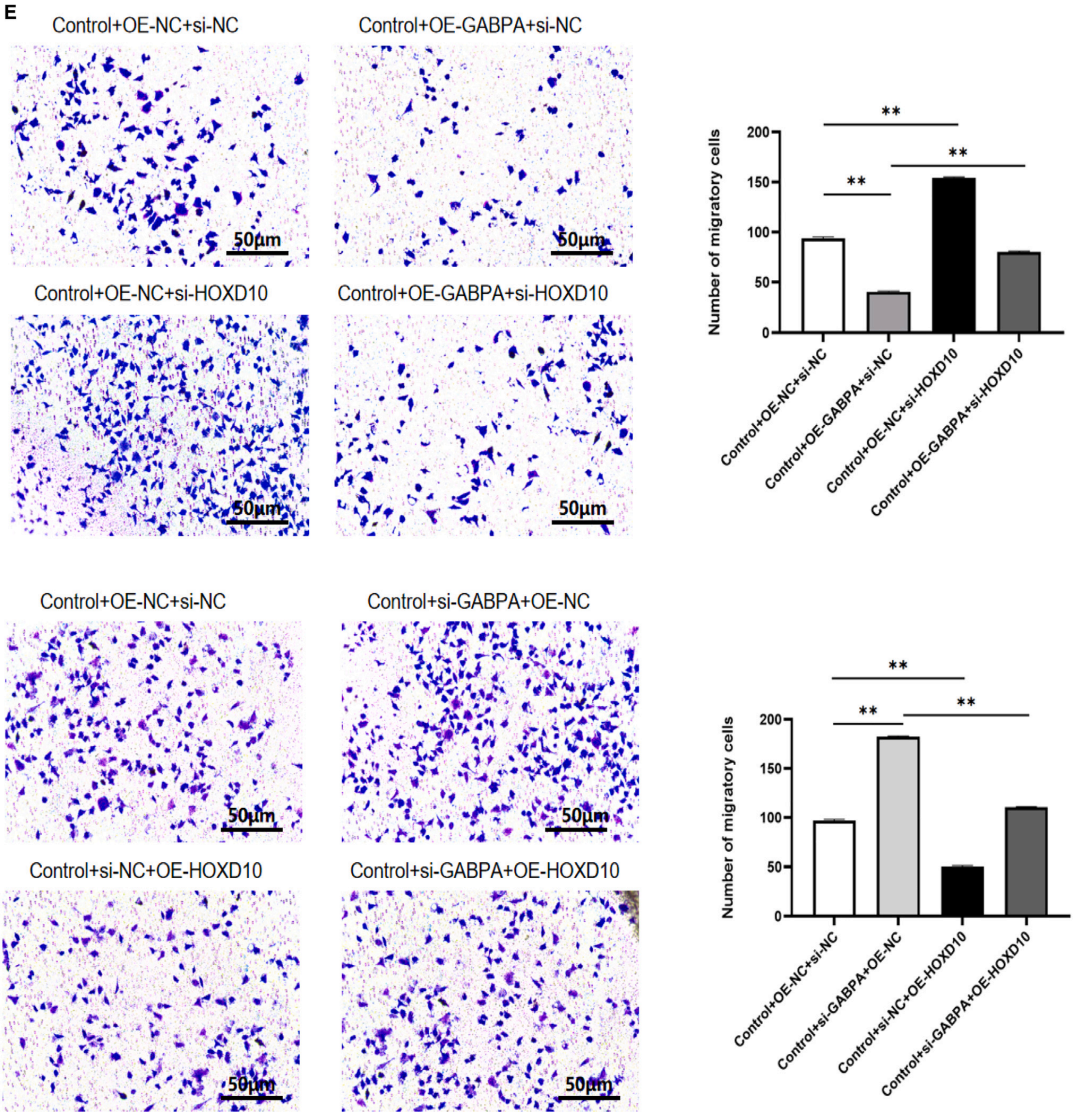
GABPA relies on HOXD10 to regulate cell behavior (A) Western blot and cellular immunofluorescence experiments showed that the expression level of HOXD10 increased after GABPA overexpression, whereas the expression level of HOXD10 decreased after GABPA knockout(∗*p* < 0.05). (B) CCK-8 assay, the proliferation of Control+OE-GABPA+si-NC group was slower than that of Control+OE-NC+si-NC group, and the proliferation of Control+OE-NC+si-HOXD10 group was faster than that of Control+OE-NC+si-NC group. The proliferation of Control+OE-GABPA+si-HOXD10 group was faster than that of Control+OE-GABPA+si-NC group. The proliferation of Control+si-GABPA+OE-NC group was accelerated compared with Control+si-NC+OE-NC group, and the proliferation of Control+si-NC+OE-HOXD10 group was slowed down compared with Control+si-NC+OE-NC group. The proliferation of Control+si-GABPA+OE-HOXD10 group was slower than that of Control+si-GABPA+OE-NC group(∗∗*p* < 0.01). (C) Control+OE-GABPA+si-NC group Annexin V positive cells increased compared with Control+OE-NC+si-NC group, and Control+OE-NC+si-HOXD10 group Annexin V positive cells decreased compared with Control+OE-NC+si-NC group. Annexin V positive cells were decreased in Control+OE-GABPA+si-HOXD10 group compared with Control+OE-GABPA+si-NC group. Annexin V positive cells decreased in the Control+si-GABPA+OE-NC group compared with the Control+si-NC+OE-NC group, Annexin V positive cells increased in the Control+si-NC+OE-HOXD10 group compared with the Control+si-NC+OE-NC group. Annexin V positive cells were increased in Control+si-GABPA+OE-HOXD10 group compared with Control+si-GABPA+OE-NC group(∗∗*p* < 0.01). (D) The number of cells entering the lower compartment in Control+OE-GABPA+si-NC group was lower than that in Control+OE-NC+si-NC group, and the number of cells entering the lower compartment in Control+OE-NC+si-HOXD10 group was higher than that in Control+OE-NC+si-NC group.More cells entered the lower compartment in Control+OE-GABPA+si-HOXD10 group than in Control+OE-GABPA+si-NC group. The number of cells entering the lower compartment was higher in Control+si-GABPA+OE-NC group than in Control+si-NC+OE-NC group, and the number of cells entering the lower compartment was lower in Control+si-NC+OE-HOXD10 group than in Control+si-NC+OE-NC group. The number of cells in Control+si-GABPA+OE-HOXD10 group was lower than that in Control+si-GABPA+OE-NC group(∗∗*p* < 0.01). (E) The number of transmembrane cells in Control+OE-GABPA+si-NC group was lower than that in Control+OE-NC+si-NC group, and the number of transmembrane cells in Control+OE-NC+si-HOXD10 group was higher than that in Control+OE-NC+si-NC group. Control+OE-GABPA+si-HOXD10 group had more transmembrane cells than Control+OE-GABPA+si-NC group. The number of transmembrane cells in Control+si-GABPA+OE-NC group was higher than that in Control+si-NC+OE-NC group, and the number of transmembrane cells in Control+si-NC+OE-HOXD10 group was lower than that in Control+si-NC+OE-NC group. The number of transmembrane cells in Control+si-GABPA+OE-HOXD10 group was lower than that in Control+si-GABPA+OE-NC group (∗∗*p* < 0.01).

To explore the role of GABPA in regulation of expression of HOXD10 in hEM15A-related phenotypes, we overexpressed GABPA and knocked down HOXD10 and vice versa. Western blotting showed that after overexpression of GABPA in hEM15A cells, expression of HOXD10 was significantly increased; the cell immunofluorescence results showed that the fluorescence intensity was higher in cells that overexpressed GABPA and that the HOXD10 expression level was significantly lower in the group with GABPA knockdown in comparison with control cells. The fluorescence intensity decreased (Figure 4A). The CCK-8 assay showed that cell proliferation was slower in the group with GABPA overexpression only and more rapid in the group with HOXD10 knockdown only in comparison with the control group. Cell proliferation was more rapid in the group with both GABA overexpression and HOXD10 knockdown than in the group with GABPA overexpression only and also more rapid in the group with GABPA knockdown only than in the control group. Cell proliferation was slower in the group with HOXD10 overexpression than in the control group and was also slower in the group with both HOXD10 overexpression and GABPA knockdown than in the group with GABPA knockdown only (Figure 4B). Annexin V/PI double staining and flow sorting showed that, compared with those in the control group, Annexin V-positive cells were greater in number in the group with GABPA overexpression only and in the group with HOXD10 knockdown only. There were fewer Annexin V-positive cells in the group with both HOXD10 overexpression and GABPA knockdown than in the group with GABPA overexpression only. Compared with the findings in the control group, there were fewer Annexin V-positive cells in the group with GABPA knockdown only and more Annexin V-positive cells in the group with HOXD10 overexpression only. Moreover, there were more Annexin V-positive cells in the group with both HOXD10 overexpression and GABPA knockdown than in the group with GABPA knockdown only (Figure 4C). The transwell assay results showed that fewer cells entered the lower compartment in the group with GABPA overexpression only than in the control group and that more cells entered the lower compartment in the group with HOXD10 knockdown only than in the control group and in the group with both GABPA overexpression and HOXD10 knockdown than in the group with GABPA overexpression only. Compared with that in the control group, the number of cells that entered the lower compartment was higher in the group with GABPA knockdown only and lower in the group with HOXD10 overexpression only. The number of cells that entered the lower compartment was also lower in the group with both HOXD10 overexpression and GABPA knockdown than in the group with GABPA knockdown only (Figure 4D). The transwell assay with Matrigel showed that there were fewer transmembrane cells in the group with GABPA overexpression only than in the control group. There were more transmembrane cells in the group with HOXD10 knockdown only than in the control group and in the group with both HOXD10 overexpression and GABPA knockdown than in the group with GABPA overexpression only. Compared with the findings in the control group, there were more transmembrane cells in the group with GABPA knockdown only and fewer transmembrane cells in the group with HOXD10 overexpression only. There were also fewer transmembrane cells in the group with both GABA knockdown and HOXD10 overexpression than in the group with GABPA knockdown only (Figure 4E). These results suggested that knockdown of HOXD10 when GABPA is overexpressed can reverse the proliferation, migration, invasion, and apoptosis of hEM15A cells induced by overexpression of GABPA. Conversely, overexpression of HOXD10 when GABPA is knocked down can reverse the proliferation, migration, invasion, and apoptosis induced by GABPA alone in these cells. These findings indicated that GABPA inhibits proliferation, migration, and invasion of hEM15A cells via transcriptional activation of HOXD10, promoting apoptosis.

### GABPA was downregulated by miR-450b-5p

**Figure 5:**
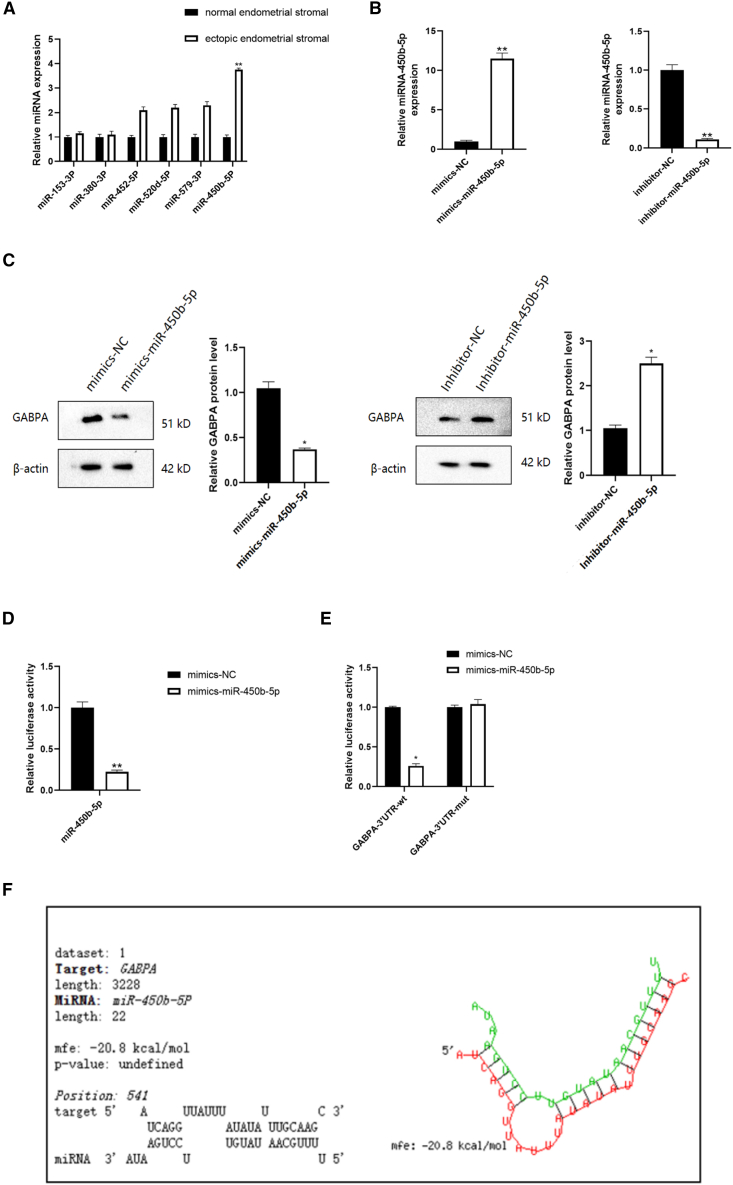
GABPA is downregulated by miR-450b-5p (A) The results of RT-qPCR showed that miR-450b-5p was highly expressed in ectopic endometrial tissues of EMS mice compared with normal control mice(∗∗*p* < 0.01). (B) The knockdown and overexpression efficiency of miR-450b-5p was detected by RT-qPCR(∗∗*p* < 0.01). (C) The effects of knockdown and overexpression of miR-450b-5p on GABPA expression were detected by western blot(∗*p* < 0.05). (D) Double luciferase reporter gene assay in HEK293T cells showed that miR-450b-5p regulates GABPA(∗∗*p* < 0.01). (E) Double luciferase reporter gene assay in hEM15A showed that miR-450b-5p regulates GABPA. (∗*p* < 0.01). (F) Specific binding sites of miR-450b-5p and GABPA-3 'UTR.

Having demonstrated that GABPA is an important inhibitory factor in EMS, we explored its upstream regulation using the ENCORI_hg38 and miRDB databases to identify the miRNAs that complement and bind to the 3′-UTR sequence of GABPA and those that potentially regulate the transcription factor GABPA. miR-450b-5p was screened for higher expression in animal tissues than the control tissues (Figure 5A). To examine the interaction between GABPA and miR-450b-5p, we first compared the expression of GABPA by adding an miR-450b-5p mimic and an miR-450b-5p inhibitor. Both western blotting and RT-qPCR showed that expression of GABPA was downregulated after addition of mimic-miR-450b-5p and upregulated after addition of inhibitor-miR-450b-5p (Figures 5B and 5C). Next, we used double luciferase reporter assays to confirm the interaction and detect whether miR-450b-5p directly binds to GABPA. The results showed that miR-450b-5p could bind to the 3′-UTR of GABPA and that addition of the mimic led to degradation of luciferase mRNA, inhibition of translation, and reduction in luciferase protein, with a corresponding decrease in the biofluorescence emitted by the luciferase-catalyzed substrates. The relative fluorescence intensity was lower in the GABPA-3′-UTR-wt + mimic-miR-450b-5p group than in the GABPA-3′-UTR-wt + NC mimic group (Figures 5D and 5E). These findings demonstrated that miR-450b-5p can inhibit GABPA and that GABPA is the direct target gene of miR-450b-5p.

### miR-450b-5p promoted growth, migration, and invasion of cells but inhibited apoptosis via GABPA

**Figure 6:**
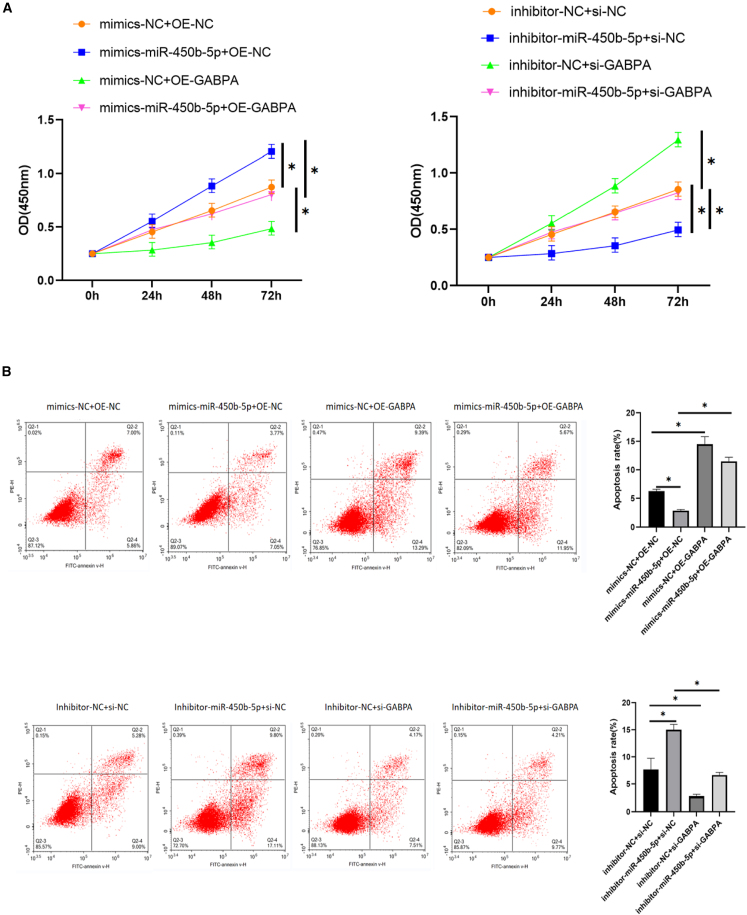

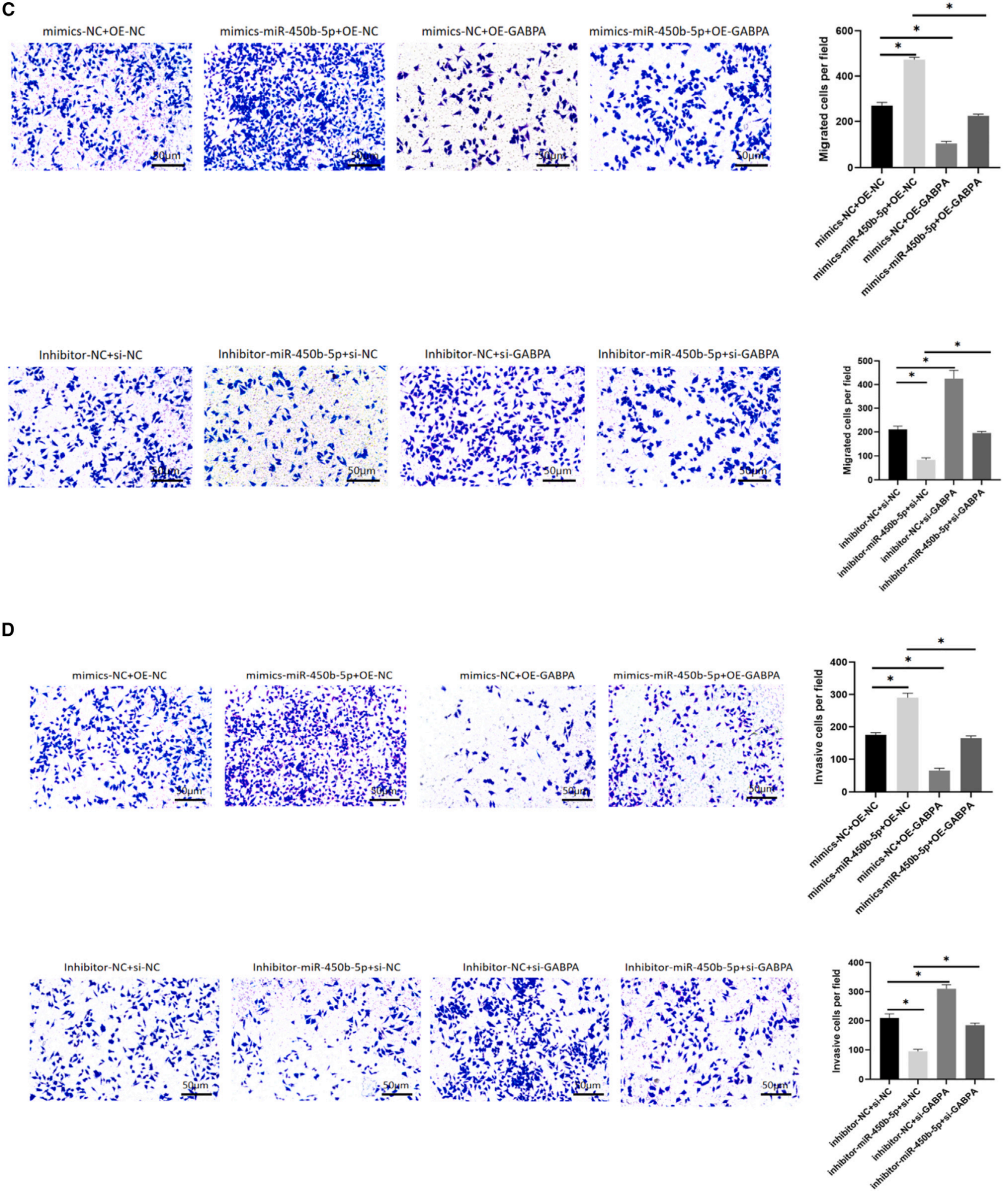
miR-450b-5p relies on GABPA to regulate hEM15A cell behavior (A) CCK-8 assay, the proliferation of mimics miR-450b-5p+OE-NC group was accelerated compared with mimics+OE-NC group, and the proliferation of mimics-NC+OE-GABPA group was slowed down compared with mimics-NC+OE-NC group. The proliferation of mimics miR-450b-5p+OE-GABPA group was slower than that of mimics miR-450b-5p+OE-NC group. Inhibitor-miR-450b-5p+si-NC group had slower proliferation than inhibitor-NC+si-NC group, and inhibitor-NC+si-GABPA group had faster proliferation than inhibitor-NC+si-NC group. Inhibitor-miR-450b-5p+si-GABPA group had faster proliferation than inhibitor-miR-450b-5p+si-NC group(∗*p* < 0.05). (B) Annexin V/PI double staining+flow sorting, the mimics miR-450b-5p+OE-NC group had lower Annexin V positive cells than the mimics+OE-NC group, and the mimics-NC+OE-GABPA group had higher Annexin V positive cells than the mimics-NC+OE-NC group. The Annexin V positive cells in miR-450b-5p+OE-GABPA group were higher than those in mimics miR-450b-5p+OE-NC group. The Annexin V positive cells in inhibitor-miR-450b-5p+si-NC group were higher than those in inhibitor-NC+si-NC group. The Annexin V positive cells in inhibitor-NC+si-GABPA group were lower than those in inhibitor-miR-450b-5p+si-NC group. The Annexin V positive cells in the inhibitor-miR-450b-5p+si-GABPA group were lower than those in the inhibitor-miR-450b-5p+si-NC group(∗*p* < 0.05). (C) Transwell migration test showed that the number of cells entering the lower chamber was higher in mimics-miR-450b-5p+OE-NC group than in mimics-NC+OE-NC group, while the number of cells entering the lower chamber was lower in mimics -NC+OE-GABPA group than in mimics-NC+OE-NC group. The number of cells entering the lower compartment in mimics miR-450b-5p+OE-GABPA group was lower than that in mimics miR-450b-5p+OE-NC group. The number of cells entering the inferior compartment was lower in inhibitor-miR-450b-5p+si-NC group than in inhibitor-NC+si-NC group group, and the number of cells entering the inferior compartment was higher in inhibitor-NC+si-GABPA group than in inhibitor-NC+si-NC group. The number of cells entering the lower compartment was more in the inhibitor-miR-450b-5p+si-GABPA group than in the inhibitor-miR-450b-5p+si-NC group(∗*p* < 0.05). (D) Invasion test showed that the number of transmembrane cells in mimics -miR-450b-5p+OE-NC group was higher than that in mimics-NC+OE-NC group, and the number of transmembrane cells in mimics-NC+OE-GABPA group was lower than that in mimics-NC+OE-NC group. The number of transmembrane cells in mimics miR-450b-5p+ OE-GABPA group was lower than that in mimics miR-450b-5p+OE-NC group. The number of penetrating cells in inhibitor-miR-450b-5p+si-NC group was lower than that in inhibitor-NC +si-NC group, and the number of penetrating cells in inhibitor-NC +si-GABPA group was higher than that in inhibitor-NC +si-NC group. The number of penetrating cells in inhibitor-miR-450B-5p +si-GABPA group was higher than that in inhibitor-miR-450B-5p +si-NC group (∗*p* < 0.05).

Next, we examined whether regulation of expression of GABPA by miR-450b-5p plays a role in promotion of EMS by miR-450b-5p. To this end, we overexpressed miR-450b-5p and knocked down GABPA and vice versa to examine whether GABPA is necessary for miR-450b-5p to be able to promote EMS. We observed the hEM15A cells for changes in proliferation, apoptosis, migration, and invasion after adding mimic-miR-450b-5p and inhibitor-miR-450b-5p and transfecting OE-GABPA and si-GABPA. CCK-8 assays indicated that proliferation was more rapid in the mimic-miR-450b-5p only group and slower in the GABPA overexpression only group when compared with that in the control group and also slower in the GABPA overexpression + mimic-miR-450b-5p group than in the mimic-miR-450b-5p only group. Compared with that in the control group, proliferation was slower in the inhibitor-miR-450b-5p only group and more rapid in the GABPA knockdown only group. Moreover, proliferation was more rapid in the inhibitor-miR-450b-5p + GABPA knockdown group than in the inhibitor-miR-450b-5p only group (Figure 6A). Annexin V/PI double staining and flow cytometry showed that there were fewer Annexin V-positive cells in the mimic-miR-450b-5p only group and in the GABPA overexpression only group than in the control group, fewer Annexin V-positive cells in the mimic-miR-450b-5p + GABPA overexpression group than in the mimic miR-450b-5p group, and fewer Annexin V-positive cells in the GABPA knockdown only group than in the control group. There were also fewer Annexin V-positive cells in the group with inhibitor-miR-450b-5p + GABPA knockdown than in the group with inhibitor-miR-450b-5p only (Figure 6B). Transwell assay results showed that more cells entered the lower chamber in the mimic-miR-450b-5p only group than in the control group and that fewer cells entered the lower chamber in the GABPA overexpression only group than in the control group. Fewer cells entered the inferior compartment in the mimic-miR-450b-5p + GABPA overexpression group than in the mimic-miR-450b-5p only group and in the mimic-miR-450b-5p only group when compared with the findings in the control group. However, more cells entered the inferior compartment in the GABPA knockdown only group than in the control group and inhibitor-miR-450b-5p + GABPA knockdown group when compared with the findings in the inhibitor-miR-450b-5p only group (Figure 6C). The transwell assay with Matrigel showed that there were more transmembrane-penetrating cells in the inhibitor-miR-450b-5p only group than in the control group. There were fewer transmembrane cells in the GABPA overexpression only group than in the control group and in the mimic-miR-450b-5p + GABPA overexpression group than in the mimic-miR-450b-5p only group. Compared with the findings in the control group, there were fewer penetrating cells in the inhibitor-miR-450b-5p only group and more penetrating cells in the GABPA knockdown only group. There were also fewer penetrating membrane cells in the inhibitor-miR-450b-5p + GABPA knockdown group than in the inhibitor-miR-450b-5p only group (Figure 6D). These results suggested that miR-450b-5p can affect proliferation, apoptosis, migration, and invasion of hEM15A cells by targeting GABPA.

### miR-450b-5p relied on GABPA to inhibit expression of HOXD10

**Figure 7:**
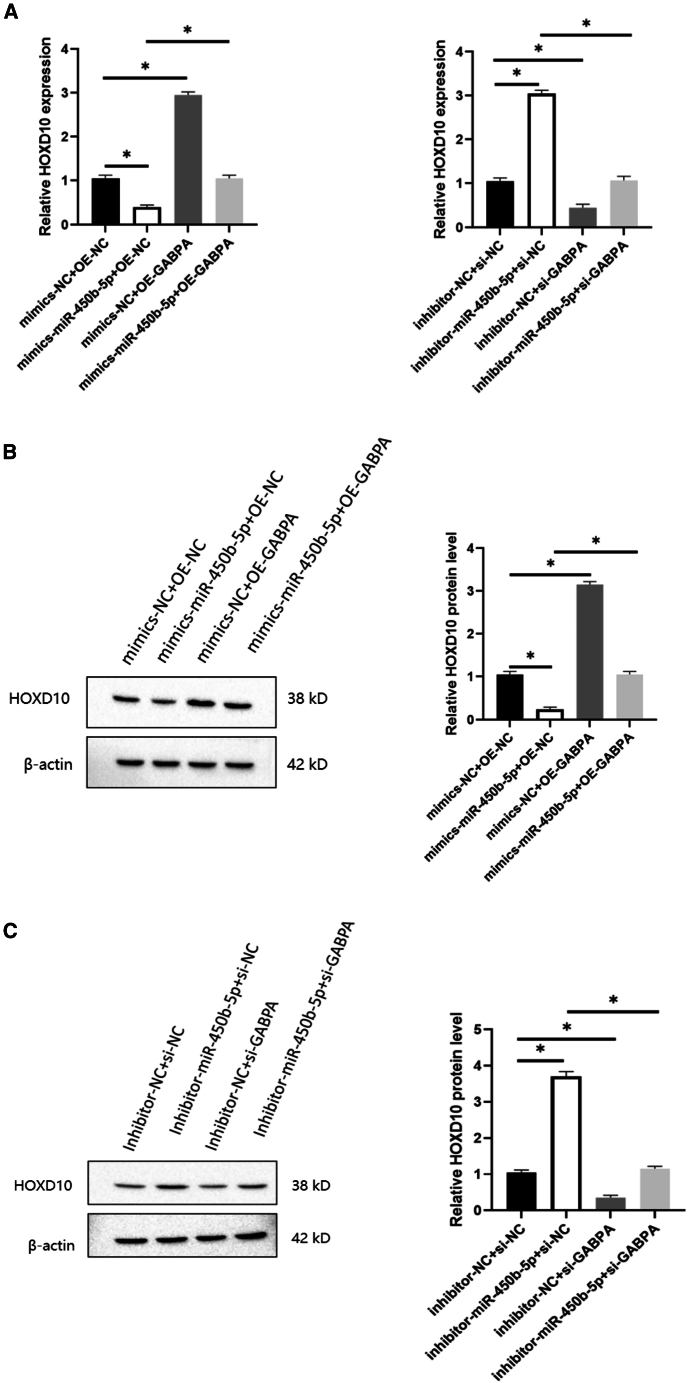
miR-450b-5p relies on GABPA to inhibit HOXD10 expression (A–C) RT-qPCR and western blot results showed that overexpression of miR-450b-5p could restore the phenotype generated by overexpression of GABPA that promoted HOXD10 expression, and knockdown of miR-450b-5p could restore the phenotype generated by GABPA that inhibited HOXD10 expression (∗*p* < 0.05).

To study the effect of GABPA on the ability of miR-450b-5p to regulate expression of HOXD10, we added a mimic and an inhibitor of miR-450b-5p to hEM15A cells and observed the expression of HOXD10 after transfection with OE-GABPA and si-GABPA. Our RT-qPCR results showed that the expression level was lower in the mimic-miR-450b-5p only group than in the control group. Conversely, expression of HOXD10 was higher in the GABPA overexpression only group than in the control group and was also higher in the mimic-miR-450b-5p + GABPA overexpression group than in the mimic-miR-450b-5p only group. The HOXD10 expression level was higher in the inhibitor-miR-450b-5p only group than in the control group and lower in the GABPA knockdown only group than in the control group. The HOXD10 expression level was also lower in the inhibitor-MiR-450B-5p + GABPA knockdown group than in the inhibitor-miR-450b-5p only group (Figure 7A). Western blotting showed that the HOXD10 expression level was lower in the mimic-miR-450b-5p only group than in the control group, higher in the GABPA overexpression only group than in the control group, higher in the mimic-miR-450b-5p + GABPA overexpression group than in the mimic-miR-450b-5p only group (Figure 7B), higher in the inhibitor-miR-450b-5p only group than in the control group, lower in the GABPA knockdown only group than in the control group, and lower in the inhibitor-miR-450B-5p + GABPA knockdown group than in the inhibitor-miR-450b-5p only group (Figure 7C).

### HOXD10 suppressed growth of endometriotic lesions *in vivo*

**Figure 8:**
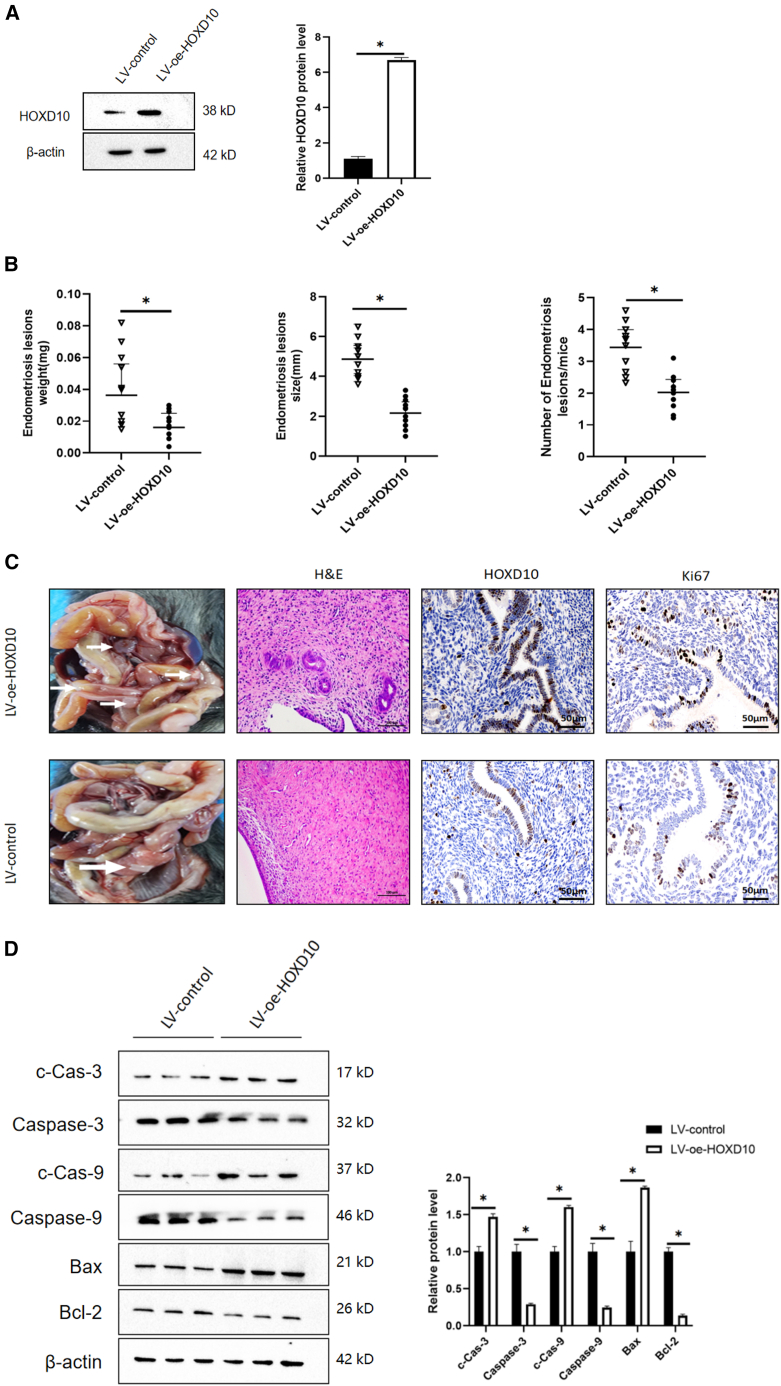
Experimental results in animal models (A) Lentivirus overexpressing HOXD10(LV-oe-HOXD10) was transfected into hEM15A, and the overexpression efficiency of lentivirus was detected by western blot (∗*p* < 0.05). (B) Recipient mice of the same genus were transplanted uterus fragments from donor mice, and the mice were randomly divided into two groups 24h after transplantation: Group 1 was a negative control group (*n* = 10), and the LV-control was injected intraperitoneally for 4 weeks; The second group, HOXD10 oe group (*n* = 10), was intraperitoneally injected with LV-oe-HOXD10 for 4 weeks. Compared with the LV-control group, the weight, size and number of lesions in the LV-oe-HOXD10 group decreased (∗*p* < 0.05). (C) The white arrow indicates ectopic endometrial tissue in an EMS mouse model. Immunohistochemistry and HE staining showed that the expression of proliferative markers in LV-oe-HOXD10 group was lower than that in LV-control group, the expression of HOXD10 was higher, and the tissue morphology returned to normal. (D) Western blot showed that the expression of pro-apoptotic molecules in LV-oe-HOXD10 group was higher than that in LV-control group, while the expression of anti-apoptotic molecules was lower in LV-oe-HOXD10 group, while the expression of anti-apoptotic molecules was higher than that in LV-control group(∗*p* < 0.05).

We developed a mouse model of EMS to investigate whether HOXD10 plays a role in progression of the disease and to determine the role of HOXD10 *in vivo*. LV-oe-HOXD10 cells, which overexpress HOXD10, were purchased from the Gemma Company (Glastonbury, CT, USA) and transfected into hEM15A cells to detect the efficiency of lentivirus (LV) overexpression. Western blotting showed that the expression level was higher in the LV-oe-HOXD10 group than in the LV control group (Figure 8A). Recipient mice underwent transplantation of uterine fragments from donor mice of the same genus. Twenty-four hours after transplantation, the mice were randomly divided into a negative control group (*n* = 10) that received intraperitoneal injections of LV for 4 weeks and an OE-HOXD10 group (*n* = 10) that received intraperitoneal injections of LV-oe-HOXD10 for 4 weeks. At the end of treatment. the weight, size, and number of endometriotic lesions were recorded. Tissue morphology, expression of HOXD10, and proliferative markers were observed by immunohistochemistry and hematoxylin-eosin (HE) staining, and apoptosis markers were detected by western blotting to evaluate the extent of apoptosis of cells in the endometriotic lesions. Measurements of lesion weight, volume, and number were lower in the LV-oe-HOXD10 group than in the LV control group (Figure 8B). The immunohistochemical and HE staining results showed lower expression levels of proliferative markers and higher HOXD10 expression levels in the LV-oe-HOXD10 group than in the LV control group with a return of tissue morphology to normal (Figure 8C). Western blotting showed that the expression levels of pro-apoptotic molecules (i.e., caspase-3, caspase-9, and Bax) were higher and those of anti-apoptotic molecules (e.g., Bcl-2) were lower in the LV-oe-HOXD10 group than in the LV control group. Compared with the findings in the LV control group, the expression levels of the three pro-apoptotic molecules were lower and the expression level of the anti-apoptotic molecule Bcl-2 was higher in the LV-oe-HOXD10 group (Figure 8D). Pathological analysis showed that stromal cells became smaller with degeneration of glandular epithelial vacuoles, a decrease in ectopic endometrial cells, and an increase in apoptosis in the model group. These findings indicated that overexpression of HOXD10 had a therapeutic effect in a mouse model of EMS.

### miR-450b-5p was negatively correlated with expression levels of GABPA and HOXD10

**Figure 9:**
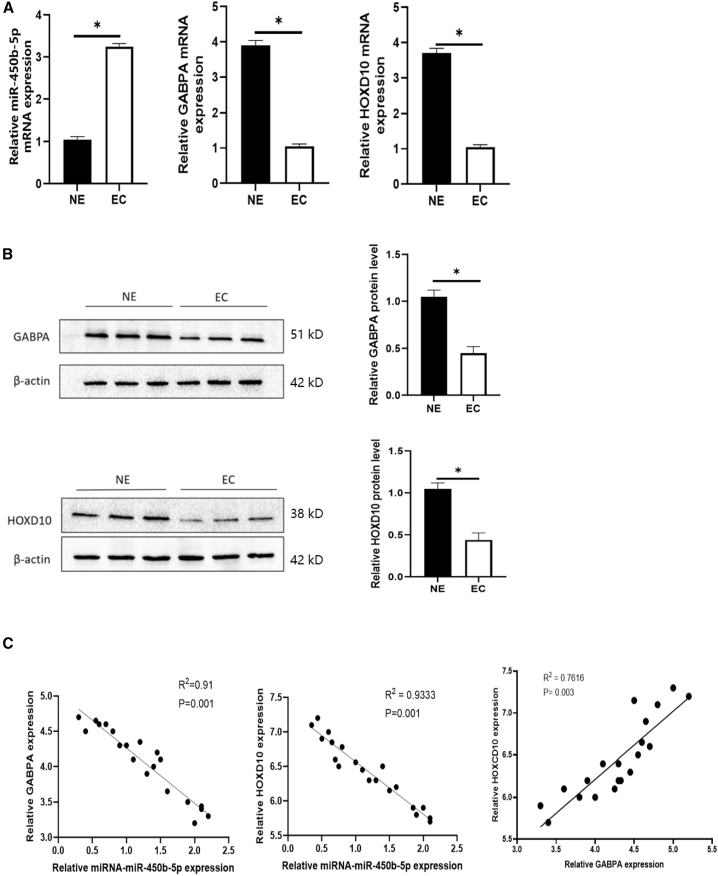
Role of miR-450b-5p in EMS (A) RT-qPCR showed that miR-450b-5p was highly expressed in the endometrial tissues of EMS patients, while GABPA was low expressed in the endometrial tissues of EMS patients(∗*p* < 0.05). (B) Western blot showed low expression of HOXD10 in endometrial tissues of EMS patients (∗*p* < 0.05). (C) The software analysis showed that the RNA level expression of miR-450b-5p and GABPA at the tissue level was negatively correlated, the protein level expression of GABPA and HOXD10 at the tissue level was positively correlated, and the RNA level expression of miR-450b-5p and HOXD10 at the tissue level was negatively correlated.

To study the function of miR-450b-5p in human EMS, we analyzed the expression level of miR-450b-5p in ectopic endometrial tissues (Figure 9A). RT-qPCR showed that the expression level of miR-450b-5p was lower in human ectopic endometrial tissue than in normal endometrial tissue. Moreover, western blotting revealed that expression levels of GABPA and HOXD10 proteins were higher in ectopic endometrial tissues than in normal endometrial tissues (Figures 9B and 9C). Furthermore, analysis of 20 samples of ectopic endometrial tissue revealed a negative correlation of GABPA and HOXD10 expression levels with the miR-450b-5p expression level. The GABPA expression level was positively correlated with the HOXD10 level (Figure 9D). Overall, these findings suggested that downregulation of miR-450b-5p and upregulation of GABPA and HOXD10 may contribute to the progression of human EMS.

## Discussion

EMS is characterized by ectopic growth of endometrial glands and stroma outside the uterine cavity, which leads to symptoms. It is a common disease among women of childbearing age, with an incidence of 10–15%. Approximately 50% of cases are associated with infertility, which has a significant impact on women’s health and quality of life. The pathogenesis of EMS is multifaceted and remains largely unresolved.^15^ There is no effective treatment for the cause, and both surgical and pharmacological treatment are associated with a high recurrence rate. Recent studies have found that the atopy of this disease is caused by the interaction between multiple gene loci and the environment, in which a change in the pelvic immune microenvironment, obstruction of the apoptosis pathway, and abnormal expression of aromatase may play important roles.^2^ MicroRNAs are a class of endogenous long non-coding RNA molecules that contain 18–22 nucleotides and appear to be involved in the pathogenesis of EMS. Despite not encoding proteins themselves, miRNAs can inhibit or switch off the expression of target genes at the post-transcriptional level by binding to the 3′-UTR of their target mRNAs. MicroRNAs are important regulatory molecules in all living organisms and have been found to be dysregulated in various types of malignancy, diabetes, and other metabolic diseases.^16^ Functional experiments have confirmed that miRNA is involved in the pathogenesis of endoheterogeneity, such as tissue hypoxia, inflammation, apoptosis, adhesion, and angiogenesis.^17^^,^^18^^,^^19^

Homeobox (HOX) genes play an important role in the growth of tumors.^20^ In a study of lung adenocarcinoma, Ma et al. identified miR-10b to be a promoter of cancer.^21^ They also found that miR-10b targeted HOXD10 and that HOXD10 was inhibited when bound to miR-10b, resulting in increased aggressiveness of tumor cells. However, expression of HOXD10 increased when antagonists were added and progression to metastasis of lung adenocarcinoma was inhibited. Myers et al. also found that sustained expression of HOXD10 can inhibit formation of new blood vessels.^22^ Conversely, when expression of HOXD10 is knocked down, the inhibitory effect on angiogenesis is weakened, increasing the aggressiveness of tumors. Studies have found a close relationship between many diseases, including cancer and cardiovascular disease, and regulation of gene expression.^23^^,^^24^^,^^25^ In-depth study of the molecular mechanism via which gene expression is regulated could provide novel ideas and methods for early diagnosis, prevention, and treatment of disease. GA-binding protein A (GABPA) is a transcription factor with a key role in development and differentiation of cells and tumorigenesis.^26^ Guo et al. demonstrated that GABPA can activate telomerase/TERT in bladder cancer and drive luminal differentiation of urinary tract epithelial cells by directly activating transcription of FOXA1 and GATA3, thereby inhibiting the aggressiveness of tumor cells and playing a tumor-suppressive role.^27^ In a series of *in vivo* and *in vitro* experiments, Zhang et al. found that GABPA inhibited the invasion and metastasis of hepatocellular carcinoma cells by partially regulating E-cadherin, thereby acting as a suppressor gene in terms of metastasis and the prognosis of the disease.^28^ However, the reasons for the different roles of GABPA in progression of different tumors and its specific mechanisms of action remain unclear. One possible explanation is that as a transcription factor, GABPA may be involved in regulation of various downstream genes; therefore, the way GABPA acts may depend on the type of tumor and its specific cellular microenvironment and signaling pathways.^29^

miRNA was initially discovered by Anelli et al. in chronic lymphocytic leukemia.^30^ They confirmed low expression of miR-15a and miR-161, thereby paving the way for investigation of miRNA in other human tumors. Further studies confirmed that miRNA is associated with many processes, including proliferation of tumors, apoptosis, and cell differentiation, and plays an important role in the development of tumors. Jin et al. found that the transcription factor TWIST can induce the transcription of miR-10b in breast cancer, and at the same time, miR-10b can affect the expression of a series of metastasis-related genes by regulating the transcription and translation of the target gene HOXD10, thereby promoting the invasion and metastasis of breast cancer.^31^ For example, miR-378 improves the survival of malignant glioma cells by reducing the activity of caspase-3, thereby promoting angiogenesis and tumor growth.^32^ Epithelial-mesenchymal transition (EMT) enables tumor cells to adapt to changes in their microenvironment, causing them to escape the tumor and enter the vascular system for metastasis and spread. miRNAs are also involved in EMT of tumor cells, whereby epithelial cells with weak diurnal polarity are transformed into stromal cells with strong depolarity *in vivo*.^33^

In previous studies, it was found that the increased expression of miR-450b-5p is associated with the enhanced inflammatory response observed in liver ischemia/reperfusion injury (IRI), which includes the upregulation of pro-inflammatory cytokines such as tumor necrosis factor-alpha (TNF-α), interleukin-1 beta (IL-1β), and interleukin-6 (IL-6). miR-450b-5p exerts its effects by targeting alpha B-crystallin (CRYAB), and the suppression of CRYAB leads to the activation of the NF-κB signaling pathway.^34^ This suggests that miR-450b-5p is involved in the body’s inflammatory response.Our study found that miR-450b-5p was abnormally expressed in ectopic endometrial tissue through previous studies. Meanwhile, bioinformatics analysis found that miR-450b-5p could act on GABPA, thereby regulating the change in activity in the signaling pathway and affecting the biological activity of endometrial cells, which has generated ideas for future research. In this study, expression of miR-450b-5p in ectopic endometrial tissue samples was detected by RT-qPCR. Subsequently, the effect of miR-450b-5p on the biological activity of endometrial cells was detected by *in vivo* and *in vitro* experiments. Using a combination of bioinformatics analysis, luciferase reporter assays, western blot assays, and additional experiments, we confirmed the expression of its target genes, clarified the role of miR-450b-5p in development of EMS, and further explored the molecular mechanism of miR-450b-5p. Our results have laid a theoretical foundation for understanding the molecular mechanism of EMS and searching for further molecular therapeutic targets.

GABPA is a transcription factor that plays a key role in development and differentiation of cells and tumorigenesis and is involved in a large number of physiological and pathological processes. Until now, there has been no research on GABPA in EMS. One study showed that GABPA can control expression of KIS, thereby inhibiting migration of vascular smooth muscle cells, which in turn affects the phosphorylation and activity of p27.^35^ In a series of *in vivo* and *in vitro* experiments by Zhang et al.,^28^ GABPA was found to partially regulate E-cadherin, thereby inhibiting the invasion and metastasis of liver cancer cells. Our study found low expression of GABPA in human ectopic endometrial tissues. Bioinformatics analysis and software prediction suggested that miR-450b-5p could target GABPA, which was confirmed in hEM15A cells. A luciferase reporter assay confirmed that miR-450b-5p can bind to the 3′-UTR of GABPA and inhibit its expression. Western blotting and RT-qPCR confirmed that miR-450b-5p could act on the 3′-UTR of GABPA and interfere with its expression at both the mRNA and protein levels.

Previous studies have shown that dysregulated expression of the HOX gene is involved in development of lung, ovarian, breast, colon, bladder, and prostate cancers.^36^ HOXD10 is a member of the HOX gene family. In renal clear cell carcinoma, HOXD10 acts as a tumor suppressor to inhibit invasion and migration of cancer cells by modulating E-cadherin and EMT.^24^

In a study by Pan et al., HOXD10 was found to activate expression of miR-7 and IGFBP3 and to lead to a biologically inhibitory phenotype, suggesting a potential therapeutic role in colorectal cancer and demonstrating that HOXD10 is frequently methylated and silenced and contributes to development of this type of cancer.^25^ In the present study, we explored the expression of HOXD10 in an hEM15A cell line and *in vivo*, changed the expression level of HOXD10 using plasmid transfection technology, and observed its effects on the proliferation, apoptosis, invasion, and migration of hEM15A cells. HOXD10 was predicted as a possible target of GABPA by software analysis and confirmed by dual luciferase reporter assay. To explore the mechanism via which HOXD10 influences the invasiveness of EMS and to provide a basis for selection of new therapeutic targets, we collected data on clinical cases to evaluate the relationship between HOXD10, GABPA, and miR-450b-5p. We used RT-qPCR and western blotting to detect the expression of HOXD10 mRNA and protein and its clinical significance in the ectopic endometrial tissue samples from 20 patients with EMS and in normal endometrial samples.

In summary, our results suggest increased expression of miR-450b-5p and decreased expression of GABPA and HOXD10 in ectopic endometrial tissues. This research has confirmed that GABPA is the direct target gene of miR-450b-5p and that GABPA protein can bind to miR-450b-5p. Overexpression of miR-450b-5p or GABPA knockdown can inhibit proliferation, migration, and invasion of cells and inhibit apoptosis. Therefore, the miR-450b-5p/GABPA/HOXD10 signaling pathway may be a potential target in the treatment of EMS.

### Limitations of the study

Our study found that miR-450b-5p is significantly overexpressed in EMS lesion tissues, affecting the proliferation, migration, invasion, and apoptosis of hEM15A cells through the modulation of the GABPA/HOXD10 axis, which plays a role in the pathogenesis and progresion of EMS. However, the research was constrained by a small sample size, comprising only 12 normal endometrial tissue samples and 12 ectopic endometrial lesion samples. Therefore, increasing the sample size is essential to validate these findings. While combining datasets from different studies can enhance classification performance, it also introduces challenges such as batch effects and variations in technical and biological aspects. Additionally, integrating multiple microarray datasets from various platforms may lead to missing values due to differing gene coverage. Future research should aim to address these limitations by expanding the sample size to further investigate the pathogenesis of EMS.

## Resource availability

### Lead contact

Further information and requests for resources and reagents should be directed to and will be fulfilled by lead contact，Yuan Yang(yangyuan0302@163.com).

### Materials availability

The study did not generate new unique materials or reagents.

### Data and code availability

• This paper does not report nucleotide sequencing-associated datasets, proteomics, peptidomics, metabolomics, structures of biological macromolecules, or small-molecule crystallography
• Any additional information about the data reported in this paper is available from the lead contact upon request.

## Acknowledgments

This study was supported by the Regional Scientists Fund of the National Institute of Science, Natural Science Foundation of China (number 82260305), the Lanzhou University 2023 Education and Teaching Reform Research Project (number 20231123), and Lanzhou University Medical Graduate Training-Obstetrics and Gynecology Professional Degree Master Graduate Management Demonstration Project (number 820809039).

## Author contributions

Y.H. and Y.Y. were mainly responsible for the conception and design of the study, acquisition of data, and drafting of the manuscript. Y.H. and Y.D.W. acquired, analyzed, and interpreted the data and drafted the manuscript. R.Y.L. and Y.M.L. contributed to the analysis and interpretation of the data and drafting of the manuscript. Y.Y. supervised the entire project, contributed to the conception and design of the study, drafted and critically revised the manuscript, approved the final version submitted for publication, and is considered the corresponding author. All the authors have read and agreed to the published version of the manuscript.

## Declaration of interests

The authors declare that they have no competing interests.

## STAR★Methods

### Key resources table

**Table:** 

REAGENT or RESOURCE	SOURCE	IDENTIFIER
10% fetal bovine serum	Gibco,USA	A5670701
TRIzol reagent	Invitrogen,USA	15596026CN
PrimeScript RT Master Mix	Takara, Japan	RR036A
Lipofectamine 3000	Invitrogen,USA	L3000015
Matrigel	Corning,USA	354230
CCK-8	Dojindo Laboratories, Japan	CK04
Annexin V-FITC kit	Life Technologies,USA	GMS10132
1% penicillin/streptomycin	Solarbio, China	P1400
Trypsin-EDTA	Solarbio, China	T1300
Anti-beta Actin	Abcam,UK	RRID: ab8226
Anti-HOXD10	Abcam,UK	RRID: ab138508
Anti-GABPA	Abcam,UK	RRID: ab224325
Anti-cleaved-capase-3	Abcam,UK	RRID: ab214430
Anti-caspase-3	Abcam,UK	RRID: ab184787
Anti-cleaved-capase-9	Cell Signaling Technology,USA	RRID: 9507S
Anti-caspase-9	Abcam,UK	RRID: ab202068
Anti-Bax	Abcam,UK	RRID: ab3191
Anti-Bcl-2	Abcam,UK	RRID: ab182858
PBS	Solarbio, China	P1020
Tris-Glycine Running Buffer	Solarbio, China	T1070
WB Transfer Buffer	Solarbio, China	D1060

### Experimental model and study participant details

#### Animals

We purchased SPF grade females from Lanzhou University Animal Laboratory Center BALB/c mice (6–8 weeks of age), all animal experiments were approved by the First Hospital of Lanzhou University Research Ethics Committee (approval number:LDYYLL2024-154).

Specific pathogen-free female BALB/c mice aged 6–8 weeks were obtained from the Laboratory Animal Center of Lanzhou University Medical School and fed for 1 week. Estradiol (100 μg/kg body weight) was injected subcutaneously into recipient and donor mice once a week until the end of the experiment. On the day of modeling, both sides of the uterine horns of the donor mouse were prepared, and medium containing PBS was added to remove fat and mesangium and other tissues. The uterus of the donor mouse was cut open and into small pieces measuring approximately 1–2 mm^3^. There were 10 recipient mice in each group, and the ratio of donor mice to recipient mice was 1:2. The uterine fragments were divided into several portions of the same weight and injected into the abdominal cavity of the recipient mice. The mice were killed 2 weeks later, and endometrial tissues of mice in the normal control group and the abdominal ectopic endometrial tissues of mice in the EMS group were collected for further experiments. The endometriosis mouse model was constructed again using the same method as above, and the mice were randomly divided into two groups after transplantation: the first was a negative control group that received intraperitoneal injection of lentivirus for 4 consecutive weeks (the LV control group) and the second was a group that received intraperitoneal injection of lentivirus overexpressing HOXD10 for 4 consecutive weeks (the LV-oe-HOXD10). The weight, quantity, and size of the lesions were measured. The lesion tissues were then stored in a refrigerator at −80°C for further experiments.

### Method details

#### Tissue collection

During laparoscopic surgery, ectopic endometrial tissue samples were collected from 12 patients with endometriosis. Control samples were taken from normal endometrial tissue from 12 women with tubal factors associated with infertility. All procedures were approved by the Human Ethics Committee of Lanzhou University First Hospital.

#### Cell culture

The hEM15A endometrial cell line was purchased from the Typical Culture Preservation Committee Cell Bank of the Chinese Academy of Sciences.The hEM15A cells

were placed in 10% fetal bovine serum (Invitrogen, San Diego, CA, USA) and 1% penicillin/streptomycin(100 U/mL) in complete culture medium. The cells were cultured at 37°C in an incubator with 5% CO_2_ saturation humidity.

#### qRT-PCR

Total RNA was extracted from hEM15A cells using TRIzol reagent (Invitrogen, Carlsbad, CA, USA) and immediately reverse-transcribed into complementary DNA using PrimeScript RT Master Mix (Takara, Shiga, Japan) according to the manufacturer’s instructions. RT-qPCR was then performed on a real-time PCR system (SureCycler 8800, Agilent Technologies, Santa Clara, CA, USA) using SYBR Premix Ex Taq II. Using GAPDH as a reference, the mRNA expression level of the target gene was calculated using the 2-ΔΔCt method.

#### Western blot assay

Total protein was extracted from hEM15A cells using radioimmunoprecipitation assay buffer and quantified using a bicinchoninic acid assay kit (Solebol, Beijing, China). Protein lysate (50 μg) was loaded onto sodium dodecyl sulfate-polyacrylamide gel for electrophoresis, after which the separated protein bands were transferred to a nitrocellulose membrane. After blocking with 5% skim milk powder at ambient temperature for 1 h, the membrane was incubated with the primary antibody at 4°C for 24 h. After washing with Tween 20-containing Tris-buffered saline, the membrane was incubated with the secondary antibody at ambient temperature for 1 h. After a final wash, the film was exposed, and the blot strips were visualized using a gel imaging system (Fusion FX5, Vilber, Collégien, France). All antibodies were purchased from Abcam (Cambridge, UK).

#### Cell transfection

The over-expressing plasmids (OE-HOXD10, 5′-TGAGGTCTCCGTGTCCAGTC-3'; OE-GABPA, 5′-AGCTTAGTGTACAGGTAATTT-3'; mimic-miR-450b-5p, 5′-GAGTCGTGCATTAAGATATTA-3′) were purchased from Shanghai Gima Pharmaceutical Technology Co. Ltd (Shanghai, China). After digesting the cells, a 6-well plate was laid, and when the cells reached 50%–60% fusion, the original medium in the 6-well plate was discarded for cell transfection. After 2–3 rinses in sterile phosphate-buffered saline (PBS), 1.5 mL of serum-free medium was added to each well. Next, 125 μL of serum-free medium and 5 μL of Lipofectamine 3000 reagent were added to a new sterile 1.5-mL EP tube and gently mixed, after which the tube was left to stand for 5 min at room temperature. We then added 125 μL of serum-free medium and 100 pmol of stay transfection sequence to another sterile 1.5-mL EP tube. The two solutions were gently mixed and allowed to stand at room temperature for 10–15 min. The mixture was added to the 6-well plate, gently shaken and mixed horizontally, and cultured in an incubator at 37°C. After 8 h, the fluid was changed to a medium containing 10% fetal bovine serum, and transfection efficiency was assessed after 24–48 h.

#### Lentivirus-constructed stable cell line

HOXD10-overexpressing lentivirus (LV-oe-HOXD10) and negative control lentivirus were sourced from Shanghai Gemma Gene Medical Technology Co., Ltd. (Shanghai, China). 293T cells in a good growth state were taken and cultured in a 10cm large dish. After the cell density reached about 30%, the packaging was started. The packaging system (Tube A: Opti-MEM 500u1, Lipo3000 15ul; Tube B: Opti-MEM 500ul, P3000 20u1, HOXD KD/OE plasmid X, PAX2 plasmid Y, MD2G plasmid Z), the mass ratio of X: Y: Z was 4:3:1. After the configuration is complete, gently blow and mix well, transfer B to A, gently blow and mix well, and leave for 15 min. Then the mixture was transferred into 293T cells that had been starved with low serum concentration for 1 h, replaced with normal medium, and continued to be placed back in the incubator. After 48 h, the venom was concentrated, and the concentrated venom was transferred into hEM15A cells to infect cells. Then the infection effect was judged by observing the GFP luminescence. The luminescence of green fluorescent protein (GFP) in cells was observed by fluorescence microscope. Finally, the infection effect was detected by qPCR experiment, and the knockdown/overexpression cell line was successfully constructed, and further follow-up experiments were conducted.

#### Dual-luciferase reporter assay

The promoter region of HOXD10 was inserted into the 5′ end of the luciferase reporter gene and denoted as HOXD10-pro-wt. Next, 1 × 10^5^ 293T cells were inoculated on 24-well plates, and 100 ng of plasmid were transfected with Lipofectamine 3000 reagent after culture for 24 h. Luciferase activity was measured 48 h after transfection using the dual luciferase reporter assay system (Promega, Madison, WI, USA) according to the manufacturer’s instructions.

#### Cell proliferation test

Cell proliferation capacity was evaluated using a CCK-8 (Dojindo Laboratories, Kumamoto, Japan) under different conditions at 24, 48, and 72 h according to the manufacturer’s instructions.

#### Flow cytometry

An Annexin V-FITC kit (Life Technologies, Waltham, MA, USA) was used for detection of apoptosis by flow cytometry according to the manufacturer’s instructions. The cell samples were analyzed using a FACScan system (BD Biosciences, Wokingham, UK). Annexin V(+)/PI(−) represents cells in early apoptosis and Annexin V(+)/PI(+) represents cells in late apoptosis.

#### Cell migration

After being starved of serum for 24 h, the cells were digested with trypsin and then centrifuged, after which the culture medium was discarded. Next, the cell density was adjusted to 1 × 10^5^/mL, and 600 μL of complete medium containing 15% fetal bovine serum were added to each lower chamber of the 24-well plate, and 200 μL of cell suspension were added to each upper chamber. The 24-well plates were cultured in a cell incubator for 24 h. The next day, the culture medium was discarded, and the cells were fixed with 4% paraformaldehyde. The fixed solution was then removed and air-dried for 10 min, after which the crystal violet-stained chamber was cleaned and air-dried using PBS. The cells were then counted under a microscope, photographed, and stored.

#### Invasiveness test

The gun head, centrifugal tube, and transwell 24-well plate were pre-cooled, and Matrigel (Corning, Corning, NY, USA) was mixed with the pre-cooled gun head. The Matrigel and serum-free medium were diluted at a ratio of 1:8, evenly spread at the bottom of the upper transwell chamber, and placed in an incubator for 3 h. After incubation, the excess liquid in the upper chamber was removed by suction. Next, 100 μL of serum-free medium were added to each well, after which the plate was placed in the incubator for 30 min to hydrate the basement membrane. The fluid in the upper chamber was removed by suction, the cells were inoculated, and the remaining steps were as described above for cell migration.

#### Hematoxylin-eosin staining

The slices were successively added to xylene I for 20 min, xylene II for 20 min, anhydrous ethanol I for 5 min, anhydrous ethanol II for 5 min, and 75% alcohol for 5 min, and then washed with water. The slices were then placed in hematoxylin dye solution for 5 min, washed, differentiated, washed, returned to blue, and then rinsed in running water. The slices were dehydrated in 85% and 95% gradient alcohol for 5 min each, and then stained with eosin dye for 5 min. The slices were successively placed into anhydrous ethanol I for 5 min, anhydrous ethanol II for 5 min, anhydrous ethanol III for 5 min, xylene I for 5 min, and xylene II for 5 min, followed by examination under a light microscope.

#### Immunohistochemistry

The tissues were fixed with 4% paraformaldehyde solution, embedded in paraffin, cut into 4-μm sections, and immunostained. The slides were soaked in xylene and ethanol for dehydration, incubated with 3% H_2_O_2_ in the dark for 25 min, and then closed with 3% bovine serum albumin at 25°C for 30 min. The slides were incubated with the primary antibody HODX10 (1:100) and Ki67 (1:100) at 37°C for 1 h. The slides were then washed and incubated with an enzyme-labeled secondary antibody at room temperature for 60 min, after which the nuclei were reverse-stained with hematoxylin for 5 min. Finally, the slides were examined under the light microscope.

#### Cell immunofluorescence

A 24-well circular climbing plate was placed into the hole of the 24-well plate, and an appropriate number of cells was placed in the 24-well plate with the climbing plate and cultured in the incubator at 37°C. The cells were removed, the medium was discarded, and the cells were washed in PBS. After the 4% paraformaldehyde was solidified, the cells were cleaned again with PBS, 0.5% Triton X-100 was added to each well, and the plate was left at room temperature for 10 min. The Triton X-100 was then discarded and the plate was cleaned with PBS. Next, 3% bovine serum albumin sealing liquid was placed into each hole. The plate was then left to stand at room temperature for 1 h, after which the sealing liquid was discarded. The diluted primary antibody was added to the tablet, which was then placed in a refrigerator at 4°C away from light and incubated overnight. On the following day, after a wash in PBS, the secondary antibody diluted with sealing solution was added to avoid light at room temperature. After incubation for 1 h, the plate was washed in PBS. DAPI working liquid was added to each hole and the plate was kept away from light for 10 min, after which it was washed in PBS. The sliver was carefully removed, the excess liquid was blotted by absorbent paper, 5 μL of anti-fluorescence quencher were added to the slide, and photographs were obtained using a confocal laser microscope.

### Quantification and statistical analysis

The experimental data in this study are summarized as the mean ± standard deviation and were plotted and analyzed using GraphPadPrism 7.0 software (GraphPad Software Inc., San Diego, CA, USA). The Student’s t test was used to compare data between the groups. A *p*-value < 0.05 was considered statistically significant.
